# Applications of Liquid-Phase Microextraction in the Sample Preparation of Environmental Solid Samples

**DOI:** 10.3390/molecules19056776

**Published:** 2014-05-23

**Authors:** Helena Prosen

**Affiliations:** Faculty of Chemistry and Chemical Technology, University of Ljubljana, Aškerčeva 5, SI-1000 Ljubljana, Slovenia; E-Mail: helena.prosen@fkkt.uni-lj.si; Tel.: +386-1-2419-176; Fax: +386-1-2419-220

**Keywords:** liquid-phase microextraction, SDME, HF-LPME, DLLME, environmental solid sample, soil, sediment

## Abstract

Solvent extraction remains one of the fundamental sample preparation techniques in the analysis of environmental solid samples, but organic solvents are toxic and environmentally harmful, therefore one of the possible greening directions is its miniaturization. The present review covers the relevant research from the field of application of microextraction to the sample preparation of environmental solid samples (soil, sediments, sewage sludge, dust *etc.*) published in the last decade. Several innovative liquid-phase microextraction (LPME) techniques that have emerged recently have also been applied as an aid in sample preparation of these samples: single-drop microextraction (SDME), hollow fiber-liquid phase microextraction (HF-LPME), dispersive liquid-liquid microextraction (DLLME). Besides the common organic solvents, surfactants and ionic liquids are also used. However, these techniques have to be combined with another technique to release the analytes from the solid sample into an aqueous solution. In the present review, the published methods were categorized into three groups: LPME in combination with a conventional solvent extraction; LPME in combination with an environmentally friendly extraction; LPME without previous extraction. The applicability of these approaches to the sample preparation for the determination of pollutants in solid environmental samples is discussed, with emphasis on their strengths, weak points and environmental impact.

## 1. Introduction

Solvent extraction (SE) remains one of the fundamental techniques employed prior to the analysis of the environmental contaminants in solid samples due to its high efficiency in transferring the compounds of interest from the frequently complex sample matrix into an analytical instrument-friendly solution. Procedures used to facilitate the transfer of analytes into the solvent are numerous: continuous extraction with a solvent at elevated temperatures (Soxhlet); mechanical or ultrasound shaking (USE); mixing of solid and solvent at elevated temperatures and pressure (pressurized liquid extraction—PLE); microwave heating and/or increased pressure (MWE); extraction with solvents with decreased viscosity, higher permeability, and higher diffusion rate, such as supercritical fluids (supercritical fluid extraction—SFE). While each of the above is a well established approach and has been demonstrated to be efficient for certain analytes, they are less advantageous for others, as has been shown in a number of critical reviews [1,2,3].

However, the main disadvantage of these techniques is the use of organic solvents. Only SFE is exempt from that, but is less widespread because of its high operating costs and complex method optimization [2]. Organic solvents are recognized as problematic for several reasons: most of them are toxic to living organisms and harmful to the environment, therefore special care has to taken for their proper disposal; moreover, they should be of high grade purity to avoid the contamination of extracts in trace analysis, and this alone significantly contributes to the high cost of analysis. These facts seem “a conflict of interest” in environmental analysis more than in any other area of analysis: analytical methods for the determination of environmental pollutants should not themselves contribute to the pollution.

To reduce its impact on the environment new developments in solvent extraction have gone in two separate directions: one is the search for more environmentally friendly solvents, the second one is miniaturization. An example of a greener solvent introduced in the environmental sample preparation is supercritical water extraction (SWE) or pressurized hot water extraction (PHWE): water at temperatures up to 650 K and sufficiently high pressure becomes less polar, less viscous and thus more applicable to the extraction of organic contaminants of lower polarity, but the disadvantages of its use are rather low extraction yields, difficulties with subsequent solvent evaporation and incompatibility with thermally unstable compounds [2,3]. Another example are ionic liquids (IL), which, although synthetic compounds, are considered more environmentally friendly because of their low volatility and lower toxicity compared to conventional organic solvents [2,3,4]. The third example is the employment of non-ionic surfactants in cloud-point extraction (CPE) or ionic surfactants in coacervative extraction (CAE) as the replacement for organic solvents [4]. Their advantages are low toxicity, low volatility and in some instances also increased biodegradability [5].

The subject of the present review is the other green direction in solvent extraction: miniaturization. Several innovative techniques have emerged, some less than a decade ago, that can be put under the umbrella of liquid-phase microextraction (LPME). The focus of the review is on the use of LPME approaches in the extraction of organic pollutants present in the solid and semi-solid environmental samples. Miniaturized solvent extractions are widely employed also to isolate different metal species in inorganic analysis, but the subject has already been covered in some excellent reviews [5,6,7]. To the best of our knowledge, there is currently no reviews dealing solely with the application of LPME to sample preparation of solid environmental samples, but there are several on the use of LPME techniques for aqueous environmental samples and the interested reader is encouraged to read them [8,9,10,11,12,13,14,15,16,17,18,19,20,21,22,23]. The emphasis of the present review is on the research published in the last decade; however, some older literature will be considered whenever necessary to facilitate the understanding of subsequent advances.

## 2. Modes and Variations of Liquid-Phase Microextraction

**Figure 1:**
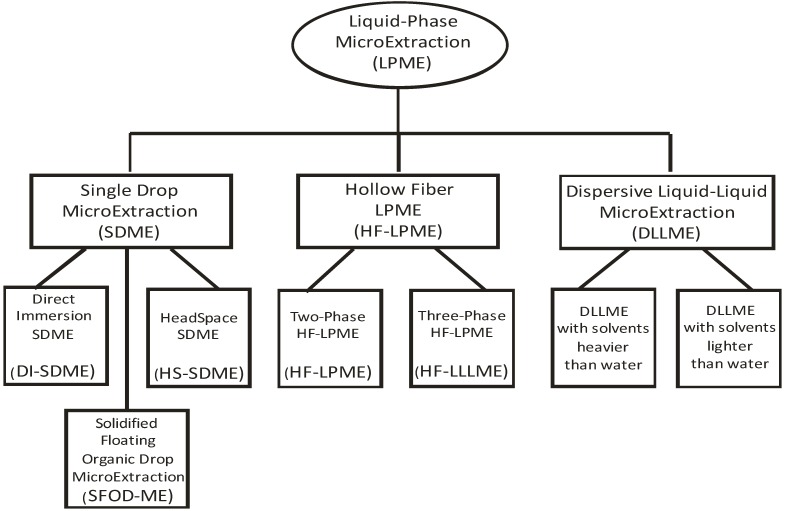
Variants of liquid-phase microextraction (LPME) with the highest number of publications.

The common feature of numerous LPME variations is the use of at least one liquid solvent, usually organic, into which the analytes of interest are extracted. The other common feature is the small volume of this solvent, typically 1–100 µL. Figure 1 is a schematic representation of the most popular modes of LPME, judging from the number of publications, deliberately leaving out the numerous subvariants. Although there is a considerable confusion with the names and abbreviations for the variants of LPME (originally named solvent microextraction, SME [24]) in the literature [25], only the currently most frequently encountered name is used throughout this paper, which may or may not originate from the inventors of the technique. 

The three main approaches to LPME are single drop microextraction (SDME), hollow fiber liquid-phase microextraction (HF-LPME) and dispersive liquid-liquid microextraction (DLLME). SDME in its original form, first described by Jeannot and Cantwell in 1996 [24], is the simplest way to perform LPME. A drop of organic solvent is exposed for a certain time to the sample, then collected and subjected to the analysis. Usually, the drop is carefully expelled from and later retracted into the syringe used in gas chromatography to inject liquid samples. A drop directly exposed to the aqueous sample is named direct immersion SDME (DI-SDME), while it can be also exposed to the headspace above the sample (HS-SDME). Alternatively, organic solvent lighter than water can be dropped to the surface of aqueous sample, left for a certain time while stirring and later collected. The latter is rather difficult to perform with a microsyringe, therefore solidification of the drop at lower temperature and its collection by spatula was designed by Zanjani *et al.* [26] and named solidified floating organic drop microextraction (SFOD-ME).

The first application of hollow fibers (HF) for the analyte extraction from aqueous samples was published by Pedersen-Bjergaard and Rasmussen [27]. In fact, their method was hollow fiber liquid-liquid-liquid microextraction (HF-LLLME). In this technique, hollow fiber (usually polypropylene) constitutes a semi-permeable membrane, in which the pores are filled with a suitable organic solvent. In the fiber lumen, the same organic solvent may be present, in this case we speak of two-phase HF-LPME, developed by the same group [28] (also: HF(2)ME). Alternatively, a different immiscible solvent is present within the fiber lumen to allow for two equilibria for the analytes: between the water and solvent in the wall; between the solvent in the wall and in the lumen; constituting the three-phase HF-LPME (HF-LLLME or HF(3)ME). 

Dispersive liquid-liquid microextraction (DLLME) is the newest addition to LPME variants, first described by Rezaee *et al.* in 2006 [29], but it has rapidly gained in popularity. A water-immiscible extraction solvent is rapidly injected into an aqueous sample together with a water-miscible disperser solvent, thus forming a very fine emulsion allowing for rapid transfer of analytes into the solvent. Solvent is subsequently separated from the sample by centrifugation.

Although these popular LPME techniques may bear some similarity, there are differences in terms of ease of optimization, ease of operation and extent of preliminary preparations, consumed time and amenability to automation. Table 1 addresses these issues, as well as some attractive modifications.

The main advantages of liquid-phase microextraction techniques are the exceptionally low consumption of organic solvents and their relative simplicity. By far the most simple is SDME, either by direct immersion or from the headspace. The instability of the drop at the tip of syringe has led researchers to propose new designs, e.g., a silicone ring at the tip of the syringe [30] or a Teflon ring design originally proposed by Ma and Cantwell in 1999 [31] to allow for back-extraction of polar compounds into an aqueous drop (liquid-liquid-liquid microextraction, LLLME). Hollow fiber-liquid phase microextraction is more cumbersome compared to SDME because of the need to prepare disposable hollow fibers, and dispersive liquid-liquid microextraction because of the need to separate the phases after the dispersion step. With extraction solvents heavier than water, a centrifugation step is needed to sediment the solvent on the bottom of a conical vial from where it is collected by a syringe; with extraction solvents lighter than water, solvent forms an upper layer and is difficult to collect by syringe only. Solidifying of the solvent (solidified floating organic drop, SFOD) is achieved by cooling the mixture in an ice bath, however this limits the choice of solvents to those with melting point of 10–30 °C. To extend the choice of solvents, special extraction vessels that facilitate the collection of the upper layer have been designed [16,17,32]. An interesting approach is demulsification of the sample-solvent emulsion in an upside-down Pasteur pipette, achieved by adding a demulsifier solvent [33]. 

**Table 1:** Properties of LPME techniques [1,3,8,9,10,11,12,13,15,16,17,18,25,32].

Technique	Solvent properties	Solvent volume	Sample preparation; other equipment	Mixing/ stirring	Extraction time	Typical analytes	Automation	Other considerations & modifications
SDME	immiscible with water; usually GC-compatible HS-SDME: low vapor pressure, also water; recent: ionic liquids	1–8 µL	GC syringe sample: filtration in DI-SDME; adjustment of ionic strength, *T*	DI-SDME: up to 600 rpm HS-SDME: higher rates	min. 1–15 min, usually longer	non-polar, semi-volatile or volatile (HS-SDME)	semi-automatic in dynamic mode; continuous flow ME (CF-ME) [34]	simple; ready-to-analyze extracts; modifications: dynamic mode possible in-needle or in-syringe; LLLME with back-extraction into droplet of 2nd immiscible solvent; exhaustive extraction by multiple HS-SDME [35]
HF-LPME	immiscible with water; compatible with HF material; low volatility & viscosity, e.g., toluene, *n*-octanol, also di-*n*-hexyl ether HF-LLLME: above valid for solvent in the HF wall; in the HF lumen: aqueous acceptor phase or ionic liquid or immiscible organic solvent	4–20 µL	small-diameter porous tube (fiber), usually polypropylene, one end sealed, other attached to syringe sample: adjustment of pH and ionic strength	vigorous stirring or vibration, microwaves	20–60 min (except for dynamic HF-LPME or EME)	non-polar; ionizable (in HF-LLLME)	yes, with autosampler; dynamic HF-LPME [36]; still each fiber manually prepared	applicable to »dirty« samples; modifications: dynamic HF-LPME [36]; solvent-bar microextraction (SBE) [37]; air in HF wall with aqueous solvent in lumen for volatile analytes [38]
DLLME	disperser solvent: miscible with water, e.g., acetone, methanol, ethanol, acetonitrile, THF; extraction solvent: ρ_solv._ > ρ_aq_, e.g., C_2_Cl_4_, Cl-benzene, CH_2_Cl_2_, CHCl_3_, CCl_4_, ionic liquid; OR ρ_solv._ < ρ_aq_, e.g., 1- or 2-dodecanol, 1-undecanol, hexadecane (*T*_mp_ ≈ room *T*), also cyclohexane, *n*-hexanol, tri-*n*-butyl-phosphate	Disp.s.: 0, 1–2 mL Extr.s.: 10–150 µL	centrifuge (ρ_solv._ > ρ_aq_); ice bath or special extracting vessel (ρ_solv._ < ρ_aq_); sample: filtration, adjustment of pH and ionic strength	not needed; ultrasound, vortex [9]	equilibration in few seconds; phase separation 1–20 min	non-polar	barely possible, although attempts [39]	modifications: temperature-controlled DLLME with ionic liquids, mixing and separation of phases at high/low *T* [40]

All variants of LPME are equilibrium techniques, and especially SDME is not exhaustive because of the small volume of extraction solvent. Attempts towards the exhaustive SDME have been made with continuous flow microextraction (CF-ME) [34] and with multiple HS-SDME [35]. However, LPME techniques are primarily applicable to extraction of non-polar analytes from aqueous samples, and thus the equilibrium is strongly shifted to the side of organic solvent. In DLLME, the partition coefficient is sometimes less favorable due to the presence of a water-miscible disperser solvent. To overcome this drawback, dispersion of extraction solvent in the sample can be achieved by application of ultrasound—US [9], vortex—VA [9,41,42] or air [43]. Alternatively, ionic liquids with aqueous solubility depending on the temperature can be used as extraction solvents in temperature-controlled IL-DLLME [40]. 

In terms of time consumption, rather long equilibration times are needed in SDME because of limited stirring rate in order to keep the drop stable; thus the process in part depends on the diffusion. Extraction time is considerably shortened in dynamic-mode SDME, both in-needle and in-syringe [44,45]. In HF-LPME, the equilibration times are even longer than in SDME, because the analytes cross the HF wall exclusively by diffusion, although more vigorous stirring of the sample can be applied in this technique. An interesting modification allowing for high stirring rates is a HF fiber filled with solvent and sealed at both ends to become a solvent bar—solvent bar microextraction (SBE) [37]. HF-LPME can also be performed in a dynamic mode [36,45,46]. A very promising modification to shorten the extraction time is the application of electric potential across the membrane in electromembrane extraction (EME), first described by Pedersen-Bjergaard and Rasmussen [47] and useful for charged analytes [8,10,48,49]. In DLLME, partitioning of the analytes into the extraction solvent is almost instantaneous because of a very fine emulsion and thus increased contact surface, therefore the time is consumed mainly during the phase separation. Extraction time becomes somewhat less important in an automated process. Attempts to automation have been made both in SDME with CF-ME [34] and in DLLME [7,39], but HF-LPME is probably the technique most amenable to automation [36,46,50].

The principal considerations for the choice of solvent are outlined in Table 1. Generally, applicable solvents are non-polar and immiscible with water, especially in SDME they should also have low volatility [51]. Rather recent additions to the choice of solvents are ionic liquids [3,13,16], which have been successfully applied to all here mentioned modes of LPME. As in the standard solvent extraction techniques, solvent is also selected on the basis of its similarity to the analytes, *i.e.*, “like dissolves like”, and its compatibility with the analytical technique applied to the determination of the analytes. By far the most extensively used technique in combination with LPME is gas chromatography (GC) with a variety of detectors. Most solvents applied in LPME are volatile enough to be amenable to GC. Exceptions are ionic liquids: an interesting solution to this problem is thermal desorption of analytes from the IL drop in the GC injector [52] or by a commercial thermodesorption system [53]. The next analytical technique frequently used for organic analytes is liquid chromatography (LC) on the reversed phase - RP [9]. Both RP-LC and capillary electrophoresis (CE) tolerate only water-miscible solvents. Several organic solvents used in LPME are too non-polar, therefore the common approach is to evaporate them, and then the dry extract is redissolved in a water-miscible solvent prior to RP-LC or CE. In inorganic analysis, LPME is usually followed by atomic absorption spectroscopy (AAS), inductively coupled plasma with optical emission spectroscopy or mass spectrometry (ICP-OES or ICP-MS) [6,7]. Applications of other analytical techniques to LPME extracts are rare, but definitely possible: UV-Vis spectrometry [20], ion-mobility spectrometry [54], X-ray fluorescence spectrometry [55] and attenuated total reflection infrared spectrometry [56].

A variety of other factors can be optimized in LPME: volume of extraction solvent, stirring rate, temperature, ionic strength and pH of the aqueous sample. In HF-LPME, fiber length determines the contact surface and volume of the extraction solvent. In DLLME, type and volume of both extraction (ES) and disperser (DS) solvent have to be optimized. 

Many of the nuances of individual liquid-phase microextraction techniques and their optimization have been left unexplored. An interested reader can find them in more exhaustive reviews on optimization and applications of SDME [22,57], HF-LPME [10,15,48], DLLME [9,13,14,16,17,18,19] or generally on LPME [8,12].

All of the LPME techniques mentioned here have been developed primarily for the extraction of analytes from aqueous samples, which belong, from the point of matrix complexity, among the samples more easily dealt with in the analytics. Aqueous samples range from the tap water with almost no interferences to the wastewater, landfill leachate and similar samples heavily loaded with interfering compounds. LPME techniques generally perform well even for these samples, although not all of them. Difficulties include clogging of hollow fiber pores or needle/syringe with particulate matter [1], dislodging of solvent drop in SDME [8,22] or adsorption of hydrophobic (macro)molecules from the sample on the surface of the hollow fiber [8]. The second problem is the interfering compounds of similar polarity as the analytes, which co-extract into solvent and complicate subsequent analysis. Due to the small volume of solvent, additional clean-up of the extract is often very difficult, although not impossible. Three-phase HF-LLLME is one of the most readily applicable solutions: unionized analyte and non-polar interferences are extracted into the hollow fiber wall, saturated with a non-polar extraction organic solvent; back-extraction of the analyte occurs into the lumen of the hollow fiber filled with acceptor solution: polar solvent [36,46,58], ionic liquid or aqueous solution of suitable pH to make the analyte ionized [27,28]. In SDME, two-phase solvent system has been used to extract and back-extract the analytes in an aqueous drop [31]. In DLLME, a technique of in-syringe back extraction has been developed by Melwanki and Fuh [59].

LPME can also be applied in the preparation of solid or semi-solid samples, but there are particular challenges because of the fragility of the approach (e.g., drop in DI-SDME [8]), disturbance of the equilibria processes, or both. Nevertheless, LPME has been applied to the sample preparation of (semi)solid biological and food samples [12,13,14,17,18,19,23], as well as environmental samples. The latter include soil, river or marine sediment, sewage sludge, dust, particulate matter in natural water samples and others. There are various approaches to include LPME in the preparation of this type of samples, and in this review, they have been grouped into three categories based on some common features.

**Table 2:** Applications of LPME on sample extracts obtained by conventional solvent extraction.

LPME	Analytical techn.	Sample	Analytes	Extraction procedure for solid sample	Optimized extraction conditions for s.s.	LPME procedure	Method performance	Ref.
DLLME	GC-ECD	soil	5 PCBs	1 g s.s. + 10 mL AC; mech. shaking 30 min; upper layer	extraction solvent	1.0 mL AC extract (DS) + 30 µL Cl-benzene (ES) inj. into 5.0 mL w.; centrif.; sedim. phase evaporated, rediss. in 20 µL *n*-hexane	η 82.3%–113.6% (3 levels); RSD < 6.4%; LOD 0.20–0.50 ng/g	[60]
US-DLLME	GC-MS (SIM)	soil	endosulfan & 5 metab.	0.5 g s.s. + 1.25 mL AC; US (10 min); centrif.	not given	AC extract (DS) + 58 μL TCE (ES) inj. into 5.0 mL w. + 7% Na_2_SO_4_; US (2 min); centrif.; direct injection	experimental design for US-DLLME optimization; η 89.0%–99.7%; RSD < 6.3%; LOD 0.316–2.494 ng/g; no interference from sample matrix observed	[61]
DLLME	HPLC-FLD	sediment	PAHs	0.2 g s.s. + 2 mL ACN, VA (2 min), centrif.	extraction solvent, vortex time	1.0 mL ACN extract (DS) + 80 μL CH_2_Cl_2_ (ES) inj. into 5.0 mL w.; centrif.; sedim. phase evaporated, rediss. in 40 μL ACN	η 72.9%–97.8% (3 levels); RSD < 8.0%; LOD 2.3–6.8 ng/g; no interference from sample matrix observed	[62]
DLLME	LC-FLD	soil	carbaryl, triazophos	1 g s.s. + 10 mL MeOH, mech. shaking (30 min); filtered	extraction solvent	1.0 mL MeOH extract (DS) + 50 µL TtCE (ES) inj. into 5.0 mL w.; centrif.; sedim. phase evaporated, rediss. in 25 μL MeOH	η 80.8%–111.1% (3 levels); RSD < 4.3%; LOD 0.014–0.110 ng/g; some matrix interferences present in chromatograms	[63]
US-IL-DLLME	HPLC-UV	soil	3 pesticides	10 g s.s. + 30 mL sol. (60% MeOH, 5 mg NaCl); US, centrif. + filtr.; repeat; evaporated to dryness, rediss. in 10 mL MeOH sol., pH adjust to 4.0	not given	1.0 mL sample sol., inj. 0.3 mL MeOH (DS) + 70 μL [BMIM]TFSI (ES); shake, US (2 min); centrif.; sedim. phase dissolved in 0.5 mL MeOH	not given for overall method (just for DLLME)	[64]
IL-DLLME	HPLC-FLD	soil	5 pesticides 2 metabol.	3 g s.s. + 20 mL MeOH + 2.5% NaCl; manual shaking + US; centrif. + filtr.; repeat; evaporated to dryness, rediss. in 10.0 mL w., pH adjust to 5.2	extraction solvent, *V*_solv_, NaCl add., US time, amount of sample	add. NaCl to 30%; MeOH (DS) + IL ([HMIm][PF_6_], ES); centrif.; 80 µL sedim. phase dissolved in 1120 µL ACN-phosphate buffer	central composite experimental design for optimization of IL-DLLME conditions; η 88%–119%; LOD 0.02–27.1 ng/g	[65]
IL-DLLME	HPLC-FLD	soil	7 pesticides and metabol.	3 g s.s. + 20 mL MeOH + 2.5% NaCl; manual shaking + US (10 min), centrif. + filtr.; repeat; evaporated to dryness, rediss. in 10.0 mL w., pH adjust to 5.2	not given	comparison of 2 IL as ES: [PPIm][PF_6_] and [HMIm][PF_6_]; add. NaCl (2.5 g); MeOH (DS) 418 μL + IL 117.5 mg (ES); VA 1 min; centrif.; 80 µL sedim. phase dissolved in 1120 µL ACN-phosphate buffer	η 93%–118%; RSD < 20%; LOD 0.02–60.5 ng/g	[66]
DLLME	sweeping MEKC-DAD	soil	5 sulfonylurea herbicides	10 g s.s. + 10 mL ACN (5% HCOOH, pH 3.0), shaking; added 4 g MgSO_4_ + 1 g NaCl, shaking; centrif.; 5 mL supernatant + 250 mg C_18_ + 1.5 g MgSO_4_, shaking; centrif., filtered	extraction solvent, pH of sample solution for DSPE, DSPE sorbent	1.0 mL extract (DS) + 50 µL ClBz inj. into 5.0 mL w. (pH 2.0, HCl), VA (5 s); centrif.; sedim. phase evaporated, rediss. in 20.0 µL phosphate buffer (pH 10.0)	η 76.0%–93.5% (3 levels); RSD < 6.8%; LOD 0.5–1.0 ng/g	[67]
DLLME	HPLC-DAD	soil	4 sulfonylurea herbicides	10 g s.s. + 20 mL AC/0.15 M NaHCO_3_ (2:8), shaking 30 min; filtered; 10 mL filtrate + 0.15 g C_18_, shaking 5 min; filtered; pH adj. to 2.0 and dil. to 25 mL with AC/w. (2:8, pH 2.0)	organic solvent, DSPE sorbent	5.0 mL solution (with AC 20% as DS) + 60 µL ClBz (ES), VA 5 s; centrif.; sedim. phase evaporated, rediss. in 15 µL ACN	η 78.0%–92.5% (3 levels); RSD < 7.2%; LOD 0.5–1.2 ng/g; some interferences present in chromatograms	[68]
DLLME	GC-MS/MS	sediment	4 PBDEs	0.25 g s.s. + 1.5 mL AC; US (35 °C) 6 × 5 min; centrif.; 1.2 mL leachate + 100 mg SiO_2_, VA (30 s); centrif.	leaching solvent, *V*_solv_, DSPE sorbent, US time & mode, US-transmitting liq., leaching *T*	1.0 mL AC extract (DS) + 60 µL CCl_4_ (ES) inj. into 5.0 mL w.; shaking, 5 min in bath (35 °C); centrif.; direct injection	η 80%–112% (2 levels); RSD < 9.8%; LOD 0.02–0.06 ng/g; extraction method comparable efficiency with Soxhlet's	[69]
DLLME	GC-MS/MS	sediment	4 PBDEs	1 g s.s. + 1.2 mL MeOH; US (40 °C) 2 × 9.2 min; centrif.	leaching solvent type (also a DS) & *V*, *T*, US time & cycles	0.1 mL MeOH (DS) + 22 mg 1-dodecanol (ES) inj. into 0.4 mL leachate + 1.0 mL 6.15 M NaCl + 4.4 mL w. at 40 °C; SFOD form. at 10 min in ice bath; collected, melt, add. 3 µL *i*-octane; direct injection	factorial (2^k^) screening & central composite design for optimization; η 71%–104% (2 levels); RSD < 9.2%; LOD 0.5–1.8 pg/g	[70]
HLLE	GC-ECD	soil	3 organo-phosph. & pyrethroid pesticides	4.0 g s.s. + 10 mL AC; mech. shaking (30 min); supernatant decanted	extraction solvent, *V*_solv_	1.0 mL AC extract (CS) + 40 µL CCl_4_ (ES) inj. into 5.0 mL w.; phase separation by 0.3 g NaCl; centrif.; direct injection	η 79.2%–113.1%; RSD < 9.6%; LOD 0.01–0.04 ng/g	[71]
USA-EME	GC-FID	soil	diazinon, chlorpyrifos	2 g s.s. + 2.5 mL MeOH; US (2 min pulse on/off); centrif., filtered	not given	1.5 mL extract + 10.5 mL w.; 14 µL toluene slowly injected during US, US (30 s); centrif.; upper phase collection, direct injection	η 90.0%–105%; RSD < 9.2%; LOD not given for soil; several interferences in the chromatogram	[72]
HF-LPME	GC-ICP-MS	soil, dust	4 PBDEs	0.5 g soil/0.05 g dust + 3 mL MeOH; US (30 min); centrif.; supernatant diluted to 10 mL with w.	not given	3.0 mL extract, 4 µL decane (ES) in 1.5-cm HF; stirring 20 min at 40 °C & 1000 rpm; direct injection	η 86.7%–110.9%; RSD < 10.4%; LOD not given for soil & dust; several interferences present in the chromatogram (brominated compounds?)	[73]
HF-LPME	GC-ECD	sediment	vinclozoline	5 g s.s. + 10 mL ACN-MeOH (9:1); US 30 min; centrif., evapor. to 0.05 mL	extraction solvent	extract + 5 mL w., 3 µL toluene (ES) in 1.3-cm HF; stirring 20 min at 800 rpm; direct injection	η 94%–96% (2 levels); RSD 6.1%; LOD 0.5 ng/g	[74]
HF-LPME	HPLC-FLD	soil	7 pesticides and metabol.	3 g s.s. + 20 mL MeOH + 2.5% NaCl; manual shaking + US (10 min); centrif. + filtr.; repeat; evaporated to dryness, rediss. in 10.0 mL w., filtered	not given	extract (pH adj. to 9.0, NaCl to 20%), 20 µL 1-octanol (ES) in 2.0-cm HF; stirring 30 min at 1440 rpm; ES evaporated, rediss. in 50 µL mobile phase for HPLC	η 85%–117%; RSD variable, up to 71% at low levels; LOD 0.001–6.94 ng/g	[75]
HF-LLLME	GC-ECD	soil	chlorophenols	2 g s.s. + 3 mL MeOH; US (2 min pulse on/off); centrif., filtered	not given except for MeOH effect on HF-LLLME	2 mL extract dil. to 20 mL with w., dodecane in 8-cm HF wall (ES) & 25 µL ACN (AS) + IS in HF lumen; stirring 30 min at 400 rpm; direct injection	η 86.3%–110%; RSD < 9.3%; LOD not given for soil	[58]
HF-LLLME, dynamic	GC-FID	soil	PAHs	2 g s.s. + 3 mL MeOH; US (2 min pulse on/off); centrif., filtered	not given except for MeOH effect on HF-LLLME	2 mL extract dil. to 20 mL with w. + dodecane in 8-cm HF wall (ES) & 25 µL ACN (AS) in HF lumen; stirring 20 min at 1000 rpm; dynamic extr. (syringe plunger); direct injection	η 84.4%–110%; RSD < 9.3%; LOD not given for soil	[36]

*Abbreviations*: **ES**—extraction solvent; **DS**—disperser solvent in DLLME; **CS**—co-solute solvent in HLLE; **AS**—acceptor solvent in HF-LLLME; **HLLE**—homogeneous LLE; **DSPE**—dispersive solid phase extraction; **IS**—internal standard; **s.s.**—solid sample. *Solvents*: **AC**—acetone; **ACN**—acetonitrile; **ClBz**—chlorobenzene; **MeOH**—methanol; **TCE**—trichloroethylene; **TtCE**—tetrachloroethane; **w.**—water (ultrapure/double deionized/MilliQ); **[HMIm][PF_6_]**—1-hexyl-3-methylimidazolium hexafluorophosphate; **[PPIm][PF_6_]**—1,3-dipentylimidazolium hexafluorophosphate.

## 3. Liquid-Phase Microextraction Applied to (Semi)Solid Environmental Samples as a Step for Preconcentration and Clean-up of Extracts

### 3.1. LPME Combined with Conventional Solvent Extraction

The first category is the extraction of the analytes from the sample with a suitable organic solvent by one of the standard extraction techniques for solid samples. After that, the extraction solvent is evaporated to dryness and the dried extract is reconstituted in an aqueous solution. LPME is then performed on this solution. Alternatively, an extract in a water-miscible organic solvent is diluted with water and LPME is performed on the resulting solution. Table 2 lists the examples of this approach.

In essence, in this approach LPME is used as a means of clean-up and additional preconcentration of the extract [25], and not as a genuine extraction technique. In some methods described in Table 2, LPME was secondary to a previous clean-up of the extract by dispersive solid-phase extraction (DSPE) [67,68,69] and therefore served only to preconcentrate the analytes. In methods where DLLME was employed directly on the extract (previously cleaned-up or not), the organic solvent used for the extraction from the solid sample was employed also as a disperser solvent [60,61,62,63,67,68,69,70]. For this reason, the overall optimization of the method included also the choice of a suitable solvent that could efficiently serve both purposes [60,62,63,67,68,69,70]. Other parameters optimized in sample extraction were sample amount [65], solvent volume [65,69,70,71], influence of ionic strength and/or pH [65], extraction time [62,65,69,70] and US cycles [69,70], as well as temperature [69,70]. In some papers, no data on sample extraction optimization is given [61,64,66,72], in most cases because some previously existing method was directly applied with a novel clean-up of the obtained extract. In all the applications listed in Table 2, DLLME parameters were extensively optimized, but the details of optimization are not given here as they are essentially the same as they would be for the aqueous samples. However, additional interfering compounds could be co-extracted from the solid sample matrix and could interfere with DLLME, e.g., by hindering the phase separation after the centrifugation step [69]. In some papers, this problem was recognized and the solvent extract had to be subjected to clean-up by DSPE [67,68,69], which eliminated most of the interfering compounds, resulting in cleaner blank chromatograms. Nevertheless, accurate quantification could be achieved only by matrix-matched calibration [69]. Another recognized problem was the presence of co-extracted humic substances that precipitated during the DLLME step [65]. However, no problems arising from the sample matrix are reported in a majority of papers applying DLLME on solvent extracts [60,61,62,63,64,66,70]. Authors generally report low detection limits, acceptable recoveries and repeatability for the spiked samples, but no study was conducted on aged residues to establish the realistic recoveries from the environmental samples. In only one study, the results of US-DSPE-DLLME extraction were compared to results of Soxhlet-solid-phase extraction (SPE) for the same samples and found to be comparable [69]. 

Two interesting LPME techniques that can be considered as variants of DLLME appear in the applications given in Table 2: homogeneous liquid-liquid extraction (HLLE) [71] employs a water-immiscible extraction solvent and a water-miscible co-solute solvent to obtain a homogeneous solution which is subsequently broken into two separate phases by adding a neutral salt (e.g., NaCl). Solvent used for the extraction of analytes from the solid sample was also used as a co-solute solvent [71]. The other technique is ultrasound-assisted emulsification microextraction (USA-EME) [72], where authors achieved emulsification of the extraction solvent in the methanol-water extract solution by ultrasound. Separated solvent (upper layer) was collected using a special centrifuge vial [72]. 

Finally, the extracts obtained by DLLME were analyzed by the chosen optimized analytical method. In some cases, the extract had to be further diluted with a suitable solvent [64,65,66] or evaporated to dryness and redissolved in a solvent compatible with the analytical method [60,62,63,67,68].

As can be seen from Table 2, the other group of LPME techniques employed for the clean-up and preconcentration of analytes from the solid sample extract is HF-LPME with two [73,74,75] or three phases [36,58]. Again, the method development emphasis in these papers has been on the optimization of HF-LPME (details not given here) and not on solvent extraction of solid samples except in one case, where different solvents were tested for their efficiency in extracting the fungicide vinclozolin from sediment samples [74]. After the extraction, the supernatant or filtrate has usually been diluted with water and subjected to HF-LPME. The residual organic solvent in the solution could adversely affect the extraction efficiency of HF-LPME, therefore some authors established the acceptable upper limit of the residual solvent [36,58]. In the method for PBDE extraction from soil and dust, the presence of residual solvent methanol in the solution was necessary to prevent the adsorption of analytes on the walls of a glass vial [73]. Only in one paper, the solvent extract was evaporated to dryness and redissolved in water before the HF-LPME [75]. Possible matrix interferences that could be co-extracted from the samples are high-molecular humic substances (HS) that could adsorb to the fiber. Lambropoulou and Albanis [74] performed HF-LPME in solutions with the addition of humic acids (HA) and ascertained that the extraction process was not affected by them, unlike in DI-SDME, where the extraction process was adversely affected already at HA concentrations above 10 mg/L [76], which are typical for natural waters.

No example of SDME application belonging to this category was found in the literature of the last decade, although it would certainly be feasible if the solid sample extract was evaporated to dryness and redissolved in water. However, matrix interferences such as humic substances would present a more prominent problem in this type of LPME [76] because of different solubility of the solvent in the presence of HS. 

In summary, solvent consumption is decreased in this approach because of LPME, but the conventional extraction techniques for solid samples still consume a considerable volume of solvent. Typical solvent consumptions for solid samples in the methods presented in Table 2 is variable: 1.2 mL to 20 mL of pure solvent per 0.2–10 g sample of soil or sediment [36,58,60,61,62,63,64,65,66,67,68,69,70,71,72,73,74,75] or 0.05 g of house dust [73]. This may seem as a small volume, but the sample size is also small and thus, the volume of solvent per mass of sample remains approximately the same as in conventional extraction methods. However, the more established procedures for the clean-up of the extracts (e.g., SPE, DSPE) require a higher volume of sample extract, and the resulting purified extract has to be evaporated to a smaller volume to achieve preconcentration. Both steps are tedious and time-consuming. Solid-phase microextraction (SPME) is a more elegant and solventless way for the clean-up of the solid samples extracts, but the organic solvent has to be eliminated beforehand. Besides, SPME fibers are certainly more expensive than a few mL of organic solvent. Overall, significant advantages in the use of LPME techniques for the clean-up and preconcentration of solvent extracts from solid environmental samples are its low cost, rather short time needed for extraction and the miniaturization of the extraction process as a whole because of the small volume of solution needed for LPME, and this fact contributes to the decrease of solvent consumption and the greening of the methods. 

**Table 3:** Applications of LPME on solid sample extracts obtained by environmentally-friendly extraction.

LPME	Analytical techn.	Sample	Analytes	Extraction procedure for solid sample	Optimized extraction conditions for s.s.	LPME procedure	Method performance	Ref.
US-DLLME	GC-ECD	soil	3 pyrethroids	MSPD: 0.1 g s.s. + 0.3 g SiO_2_ (*d* 38 µm) blended in mortar; transf. to cartridge with 0.1 g Na_2_SO_4(anhyd)_; eluted with 3 mL AC; evaporated to 0.5 mL	sorbent, sample/sorb. ratio, eluting solv. type and *V*,	AC extract (DS) + 50 µL TtCEt (ES) inj. into 5 mL w.; US 2 min; centrif.; sedim. phase evaporated, rediss. in 20 µL *n*-hexane	η 83.6%–98.5%; RSD < 7.3%; LOD 0.45–1.13 ng/g	[77]
DLLME	HPLC-FLD	soil	carbendazime thiabendazole	20 g s.s. + 40 mL 0.1 mol/L HCl; mech. shaking 30 min; filtered, pH adj. to 7.0	not given	0.75 mL THF (DS) + 80 µL CHCl_3_ (ES) inj. into 5.0 mL solution + 0.5 g NaCl; centrif.; sedim. phase evaporated, rediss. in 15 µL MeOH	η 82.0%–93.4% (2 levels); RSD < 7.3%; LOD 1.0–1.6 ng/g	[78]
DLLME	GC-FID	sediment	PAHs	SFE: 1.2 g s.s. + 50 µL MeOH (PM); SFE at *T* 313 K, *p* 253.2 bar, static *t*_extr_ 10 min, dynamic *t*_extr_ 30 min, CO_2_ *F* = 0.5 mL/min; collected in 1 mL ACN in ice bath	pressure, temperature, static & dynamic extraction time	1.0 mL extract (DS) + 16 µL ClBz (ES) inj. into 5 mL w.; centrif.; direct injection	η 67.8%–98.9%; RSD < 10.3%; LOD 200 ng/g	[79]
DLLME	GC-FID	soil sediment	7 organo-phosphor. pesticides	SFE: 1.2 g s.s. . + 50 µL MeOH (PM); SFE at *T* 60 °C, *p* 150 bar, static *t*_extr_ 10 min, dynamic *t*_extr_ 30 min, CO_2_ *F* = 0.5 mL/min; collected in 1 mL ACN in ice bath	pressure, temperature, static & dynamic extraction time	1.0 mL extract (DS) + 17 µL CCl_4_ (ES) inj. into 5 mL w.; centrif.; direct injection	η 80%–100%; RSD < 75%; LOD 1–9 ng/g	[80]
DLLME	GC-FID	soil	2 nitrotoluenes	SFE: 2 g s.s. + 150 µL MeOH (PM); SFE at *T* 35 °C, *p* 350 atm, static *t*_extr_ 10 min, dynamic *t*_extr_ 30 min, CO_2_ *F* = 0.4 mL/min; collected in 1 mL MeOH in ice bath	central composite design to optimize SFE parameters: *T*, pressure, *V*_PM_, dynamic *t*_extr_	1.0 mL extract (DS) + 20 µL CCl_4_ (ES) inj. into 5.0 mL w. (3% NaCl); centrif.; direct injection	η 80%–84%; RSD < 6.5%; LOD 0.12 µg/g	[81]
DLLME	GC-MS	sediment	hydroxylated PAHs	SWE: 10 g s.s. + 2 g diatomaceous earth; PLE with w. pH 3.0 + 20% ACN (OM) 10 min at 150 °C & 1500 psi; purged with N_2_, collected 11 mL extract	type and *V* of organic modifier for SWE, pH, *T*, pressure, extr. time	100 µL ClBz (ES) inj. into 11 mL extract (20% ACN as DS); VA 30 s; centrif.; sedim. phase evaporated, added 50 µL MTBSTFA to derivatize, evaporated, rediss. in 100 µL AC	η 57.63%–91.07%; RSD < 11.07%; LOD 0.0139–0.2334 ng/g; comparison with SWE-SPE - all parameters better for SWE-DLLME	[82]
DLLME	GC-MS	pyrolysis solid residue	15 aromatic volatiles	extracted s.s. (extr. with CH_2_Cl_2_) and raw s.s. leached with 0.001 M CaCl_2_ sol. (leach test ISO/TS 21268-2)	not given	0.5 mL AC (DS) + 50 µL CCl_4_ (ES) inj. into 5.0 mL leachate; centrif.; direct injection	LOD 1.02–24.6 ng/L ^a^; compared with static HS and HS-SPME (both lower LODs)	[83]
DLLME	GC-MS	pyrolysis solid residue	11 alkylphenols	extracted s.s. (extr. with CH_2_Cl_2_) and raw s.s. leached with 0.001 M CaCl_2_ sol. (leach test ISO/TS 21268-2)	not given	1.0 mL AC (DS) + 15 µL TtCEt (ES) inj. into 4.0 mL leachate + NaCl (15%); centrif.; direct injection	η 61.9%–101.4%; RSD < 8.0%; LOD 0.07–0.17 µg/L ^b^	[84]
DLLME	GC-MS	particul. matter in seawater	8 UV filters	unfiltered seawater, US 15 min; pH adj. to 2.5 with acetic a.; filtered	US time	250 µL AC (DS) + 50 µL CHCl_3_ (ES) inj. into 5.0 mL sample; centrif.; direct injection	η 88%–117% (2 levels); RSD < 14%; LOD 10–30 ng/L ^a^	[85]
DLLME & in-syringe back-extract.	HPLC-UV	soil sediment	5 chlorophenols	MWE: 1.2 g s.s. + 2 mL w. (pH 10.0); MWE 90 s, cooling, diluted to 5 mL with w., pH adj. to 6.0; centrif., filtered	*V*_solv_, pH_solv_, MWE time	1.0 mL AC (DS) + 37 µL ClBz (ES) inj. into 5.0 mL extract; centrif.; 20 µL sedim. phase in syringe, then 20 µL w. (pH 12.0), plunger moving 5 min; w. phase injected	η 66.1%–82.0%; RSD < 7.6%; LOD 0.5–2.0 ng/g; chromatograms free of interferences	[86]
USA-EME	HPLC-DAD	soil	triazine herbicides	10 g s.s. + 10 mL w.; mech. shaking 40 min; filtered, diluted to 10.0 mL with w.	not given	5.0 mL extract + 100 µL ClBz,; US 3 min at 25 °C; centrif.; sedim. phase evaporated, rediss. in 20 µL MeOH	η 82.6%–92% (2 levels); RSD < 4.3%; LOD 0.1–0.5 ng/g	[87]
ATPS	HPLC-UV	soil	2 phytohormones	10 g s.s. + 30 mL MeOH/w. (80:20); US 20 min; centrif.; repeat; filtered, evaporated, rediss. in 10 mL MeOH/w. (80:20, pH 3)	not given	1.0 mL solution + 0.6 g [BMIM]Br + 0.75 g K_2_HPO_4_; stirred 10 min at 30 °C; centrif.; upper phase collected, direct injection	η 86%–102%; RSD < 5.3%; LOD 2–10 ng/g; compared to direct HF-LPME	[88]
CAE-ME	HPLC-DAD	sediment	PAHs, alkyl-phenols, paraben	MWE: 0.1 g s.s. + 3 mL 40 mM CTAB solution; MWE for 6 min at 90 °C and 140 W, cooled; centrif., filtered	*T*, MW power, CTAB solution *V* and concentration	2 mL solution + 200 µL ACN + 46 µL Li-NTf_2_ 0.5 g/mL; VA 3 min; heated 2 min at 65 °C; centrif.; sedimented droplet dil. to 100 µL with ACN, VA	η 92.8%–95.7% (2 levels); RSD < 19.3%; LOQ 0.02–0.36 µg/g; several interferences from the sample co-extracted	[89]
*in-situ* LPME with IL-based surfactant	HPLC-DAD	sediment	PAHs, alkyl-phenols, paraben	MWE: 0.1 g s.s. + 3–5 mL 40 mM C_16_MIm-Br sol.; MWE for 6 min at 90 °C and 140 W, cooled; centrif., filtered	*T*, type of ILS, ILS solution *V* and concentration	4 mL solution + 800 µL ACN + 92 µL Li-NTf_2_ 0.5 g/mL; heated 5 min at 65 °C; VA 3 min; centrif.; sedimented droplet (≈90 µL) dil. to 200 µL with ACN, VA	η (2 levels) 91.1%–127%; RSD < 19%; LOQ 0.04–1.0 µg/g	[90]
HF-LPME; DLLME	GC-FPD	soil	6 organosulfur pesticides	5 g s.s. + 10 mL w.; US 40 min; centrif.; used for HF-LPME or filtered (2×), diluted 25× with w. for DLLME	not given	HF-LPME: 5.0 mL extract, 5 µL *o*-xylene (ES) in 1-cm HF; stirring 35 min at 1200 rpm; direct injectionDLLME: 0.8 mL MeOH (DS) + 10 µL CCl_4_ (ES) inj. into 5.0 mL solution; centrif.; direct injection	HF-LPME: η 81.7%–109.2%; RSD < 9.6%; DLLME: η 87.8%–100.6%; RSD < 9.0%; LOD not given for soil samples Comparison: DLLME faster & higher capacity, HF-LPME more robust & simple for complex samples	[91]
HF-LPME	GC-MS	sediment	12 OCPs 8 PCBs	MWE: 1 g s.s. + 10 mL w.; MWE at 600 W for 20 min at 80 °C; supernatant diluted to 10 mL	*T*, extraction time	10 mL extract, 5 µL toluene (ES) in 1.3-cm HF; stirring 20 min at 700 rpm; direct injection	η 73%–111% (OCP) 86–110 % (PCB); RSD < 20%; LOD 0.07–0.70 ng/g	[92]
HF-LLLME	LC-ESI-MS	dried sewage sludge	NSAIDs	PHWE: 0.5 g s.s. + 20 g sea sand, PLE with 0.01 M NaOH 5 min (5 cyc.) at 120 °C & 100 bar, flush *V* 60 %; purged with N_2_, collected 90 mL extract adj. pH to 1.5 and diluted to 100 mL	pH of solvent, *T*, number of cycles, flush volume	100 mL extract, DHE in 10-cm HF wall (ES) & 25 µL 0.1 M (NH_4_)_2_CO_3_ (AS) in HF lumen; stirring 120 min at 600 rpm; direct injection	η (PHWE) 101%–109% (spike), 38.9%–90.3%(native); η (HF-LPME) 23.6%–30.3%; RSD < 20%; LOD 0.4–3.7 ng/g; only small matrix effect in ESI	[93]
HF-LLLME	LC-ESI-MS	dried sewage sludge	SSRIs	PHWE: 0.5 g s.s. + 20 g sea sand; PLE with 0.05 M H_3_PO_4_ pH 2 for 5 min (5 cyc.) at 120 °C & 100 bar, flush *V* 90%; purged with N_2_, collected 90 mL extract adj. pH to 12.4 and diluted to 100 mL	pH of solvent, *T*, number of cycles, flush volume	100 mL extract, DHE in 10-cm HF wall (ES) & 0.1 M (NH_4_)H_2_PO_4_ pH 2.1 (AS) in HF lumen; stirring 8 h; direct injection	η (PHWE) 67%–83%(spike) 72.2%–85.8%(native); η (HF-LPME) 29%–47%; RSD < 20.8 %; LOD 6 ng/g; comparison to direct HF-LLLME method (without PHWE)	[94]
HF-LLLME	LC-MS/MS	sewage sludge	SSRIs and metabolites	1 g s.s. + 1.1 L w. + 20 µL HCOOH; stirred 16 h at 900 rpm; filtered, diluted 1:100 or 1:20	not given	solution + IS + 10 mL 5 M NaOH, DHE in 28-cm HF wall (ES) & 20 µL w.+HCOOH pH 2 (AS) in HF lumen; stirring 2 h at 800 rpm; direct injection	η 26.2%–71.4%; RSD < 24.6%(SSRI), < 51% (metab.); LOD not given	[95]
DI-SDME	AP-MALDI-MS	soil	antibiotic monensin	5 g s.s. + 15 mL w. (10% NaCl); shaking 5 min, US 5 min; centrif., repeat; supernatants collected	not given	20.0 mL solution + 10% NaCl, 1.5 µL CHCl_3_/toluene (1:1) drop immersed for 10 min at 240 rpm; direct injection	η 74.5%–82.8% (3 levels); RSD < 6.5%; LOD 12.4 ng/mL ^b^	[96]
HS-SDME	GC-FID	fire debris	fire accelerants	20x20 cm piece of textile soaked with accelerant, ignited; debris + 100 mL w., mixed 3 min; centrif., filtered	sample volume	10 mL filtrate stirred at 1500 rpm, 2.5 µL benzyl alcohol drop exposed to HS for 20 min; direct injection	LOD 0.15 mg/L ^a^	[97]
ESy	GC-ECD GC-MS	soil	OCPs	1 g s.s. + 10 mL w./ACN (8:2); US 15 min; centrif.; supernatant + 70 µL conc. H_3_PO_4_ + 100 mg Cu granules; US 15 min; filtered	ACN addition to extr. solvent	3 mL filtrate flushed through donor side ESy at 100 µL/min; acceptor phase: *n*-undecane; direct injection	compared with SE and PLE: comparable results, less solvent (~4 mL *vs.* 420 mL-SE or 18 mL-PLE) and time (1.5 h *vs.* 4 h-SE or 0.85 h-PLE), less s.s.	[98]

^a^ given in liquid sample/leachate/filtrate; ^b ^ LOD for soil given in ng/mL. *Abbreviations*: **ES**—extraction solvent; **DS**—disperser solvent in DLLME; **AS**—acceptor solvent in HF-LLLME; **PM**—polar modifier in SFE; **OM**—organic modifier in PLE; **ATPS**—aqueous two-phase system; **ESy**—extracting syringe; **MSPD**—matrix solid phase dispersion; **s.s.**—solid sample. *Solvents*: **AC**—acetone; **ACN**—acetonitrile; **ClBz**—chlorobenzene; **DHE**—di-*n*-hexyl ether; **ILS**—ionic liquid-based surfactant, **MeOH**—methanol; **THF**—tetrahydrofuran; **TtCEt**—tetrachloroethylene; **w.**—water (ultrapure/double deionized/MilliQ). *Reagents*: **CTAB**—cetyltrimethylammonium bromide; **Li-NTf_2_**—lithium bis[(trifluoromethane)sulfonyl]imide; **MTBSTFA**—*N*-(tert-butyldimethylsilyl)-*N*-methyl-trifluoroacetamide; **[BMIm]Br**—1-butyl-3-methylimidazolium bromide; **C_16_MIm-Br**—1-hexadecyl-3-methylimidazolium bromide.

### 3.2. LPME Combined with Environmentally-Friendly Extraction

The second category of LPME applications for solid environmental samples also employs an extraction technique before LPME, but in this case, more environmentally friendly solvents are used: supercritical CO_2_ in SFE; water in SWE or PHWE; aqueous solutions, ionic liquids or surfactants as extraction solvents in combination with ultrasound or microwaves. LPME technique is necessary as a step to preconcentrate the analytes from the aqueous extract. Examples are given in Table 3.

As in the first category, LPME techniques are used as a means to clean-up and preconcentrate the extracts of solid samples, but these extracts are now prepared with environmentally friendly extraction methods. One frequent feature of the published methods, compared to the previous category, is the more extensive optimization of extraction parameters.

Supercritical fluid extraction (SFE) was used to extract pollutants of various polarities (PAHs, organophosphorus pesticides, nitrotoluenes) from sediment and soil samples [79,80,81]. With this extraction method, a small volume of polar modifier, *i.e.*, organic solvent, is added to supercritical CO_2_. In all three published methods, 50–150 µL of methanol per extract was used for this purpose [79,80,81]. However, the final SFE extract was collected in 1 mL of acetonitrile [79,80] or methanol [81]. The LPME technique following SFE was DLLME, therefore the collecting solvent essentially served as a disperser solvent in DLLME as well [79,80,81]. The overall consumption of organic solvent per sample was thus no higher than in the case of DLLME of an aqueous sample. In all methods, SFE was optimized in terms of pressure, temperature, static and dynamic extraction time [79,80,81], and also volume of polar modifier [81]. Since SFE can be optimized to provide the best selectivity for analyte extraction from the sample matrix, there are no reports of any interfering compound being co-extracted. Performance of the extraction method was satisfactory in all papers. Rezaee *et al.* [79] compared their SFE-DLLME procedure to Soxhlet extraction. Recoveries were comparable, but the consumption of solvents and time was greatly diminished in SFE-DLLME.

Most methods listed in Table 3, however, employ water or a mixture of water and a water-miscible organic solvent to extract the pollutants. Supercritical water extraction (SWE)/pressurized hot water extraction (PHWE) was performed as PLE in a commercial ASE^®^ apparatus [82,93,94]. Also in this method, a certain amount (up to 20%) of organic modifier—acetonitrile—could be of benefit to the extraction efficiency [82], but was not absolutely necessary for other analytes [93,94]. Organic modifier also served as a disperser solvent in the subsequent DLLME [82]. One of the main parameters affecting the extraction yield is the pH of the extraction solution, which was optimized in all cases [82,93,94], besides the temperature [82,93,94], pressure, extraction time, type and volume of organic modifier [82] or number of extraction cycles and the final flush volume [93,94]. Pressure of the supercritical water could be the decisive factor in the co-extraction of interfering compounds, yet it was not optimized in all methods. Higher pressure means more dissolved organic matter, which interferes with DLLME process, resulting in its lower efficiency [82]. In two methods published by the same research group [93,94], the subsequent LPME technique was HF-LLLME. In the acceptor solvent in the fiber lumen, as well as in PHW before that, pH was adjusted to a similar value to promote the transfer of analytes into the solvent: alkaline pH in the extraction of non-steroidal anti-inflammatory drugs (NSAIDs) from sewage sludge [93] or acidic pH cca. 2 in the case of extraction of selective serotonin reuptake inhibitors (SSRIs) from the sewage sludge [94].

Another extraction technique employed is microwave-assisted extraction (MWE) [86,89,90,92]. Water with suitably adjusted pH was used as an extraction solvent [86,92] and its volume and pH [86], as well as extraction time [86,92] and temperature [92] were optimized. HF-LPME was subsequently applied [92] or DLLME with innovative in-syringe back-extraction from a thin layer of DLLME sedimented phase into water with alkaline pH [86]. 

In all other methods described in Table 3 where an aqueous solution was used as an extraction solvent, ultrasound or mechanical shaking/stirring [78,83,84,87,95,97] were applied to facilitate the extraction of analytes. In many methods, no data on the extraction optimization are given [78,83,84,87,91,95,96]. In others, the following parameters were optimized: ultrasound time [85], sample volume [97] or the addition of organic solvent to water [98]. On the sample extract, DLLME was applied [78,83,84,85,91], also HF-LPME [91], HF-LLLME [95], DI-SDME [96] or HS-SDME [97]. Xiong and Hu [91] compared HF-LPME and DLLME for the same US aqueous extract of organosulfur pesticides from the soil. Methods were comparable in terms of recovery and precision, but DLLME was found to be faster and had a higher capacity, while HF-LPME was more robust and simpler to employ on complex samples [91]. Wu *et al.* [87] applied USA-EME on the aqueous extract of triazines from the soil. An innovative approach named Extracting Syringe (ESy) was developed by Barri *et al.* [98]: an aqueous donor solution of analytes was flushed through a microchannel and analytes were extracted through a membrane into non-polar solvent present in the opposite microchannel. The process was automated, method performance was comparable with solvent extraction or PLE, but with decreased solvent and sample consumption and decreased or comparable extraction time [98].

Dong *et al.* [88] proposed an aqueous two-phase system (ATPS) following US-assisted extraction of phytohormones from the soil by a mixture of methanol and water. In ATPS, ionic liquid was added to the methanol-water solution and the phases were separated following the addition of an inorganic salt. Method had an excellent performance and was compared with direct HF-LPME with a similar IL in a solvent bar for extraction from the soil suspension in NaCl solution, which gave slightly poorer results in terms of recoveries and LODs; however, no extra solvent except IL was needed in HF-LPME, while 32 mL of methanol per soil sample were consumed in ATPS [88].

A common cationic surfactant (CTAB) solution [89] or an IL-based surfactant solution [90] were employed by the same research group [89,90] in combination with MWE to extract PAHs, alkylphenols and a paraben from the sediment. Various parameters were optimized: temperature, concentration and volume of surfactant solution [89,90], MW power [89] and type of IL-based surfactant [90]. In the obtained filtrate, surfactant was made insoluble by the addition of an anion-exchange reagent lithium bis[(trifluoromethane)sulfonyl]imide and acetonitrile. After centrifugation, analyte-rich sedimented phase was diluted with acetonitrile to decrease viscosity and subjected to HPLC-DAD analysis. Method performance was very good in both cases [89,90], but in the method employing CTAB several interfering compounds co-extracted from the sediment and were visible in the chromatogram [89].

Matrix solid-phase dispersion (MSPD) is an extraction approach for solid samples in which sorbent and sample are blended together in a mortar; the mixture is then transferred to an empty cartridge and the analytes eluted with a suitable solvent. It was employed by Wang *et al.* [77] to extract pyrethroid insecticides from the soil; analytes were eluted with acetonitrile, which served as a disperser solvent in subsequent US-DLLME. Type of sorbent, sorbent/sample ratio, eluting solvent type and volume were optimized [77].

As seen from Table 3 and the above discussion, no details on LPME optimization are given here except where some innovative or uncommon approaches were adopted. Otherwise, optimization of DLLME, HF-LPME and SDME parameters in the above methods was the same as for aqueous samples. The obtained extracts were analyzed by GC or HPLC in all but one method [96]. In some cases, evaporation of extract and redissolution in different solvent [77,78,[87], dilution [89,90] or even derivatization [82] was needed for the sake of compatibility with the analytical method.

All three types of LPME were generally reported to perform well on aqueous extracts of solid samples. Again, DLLME and its variants were the most frequently used approach [77,78,79,80,81,82,83,84,85,86,87,88,89,90,91], followed by HF-LPME [91,92,93,94,95] and SDME [96,97]. In some papers, authors compared the developed LPME procedure for the chosen analytes with another method on the basis of a more established extraction technique: SWE-SPE [82], static HS [83], HS-SPME [83], solvent extraction [98] or PLE [98]. Generally, LPME combined with environmentally-friendly sample extraction provided equally good or better results. An exception was determination of aromatic volatiles in pyrolysis leachate. Static headspace or HS-SPME provided better LODs and extracted less interfering compounds because of better specificity for volatile analytes compared to DLLME [83]. Although water or buffer solutions are generally deemed not entirely suitable to extract less polar analytes [2,3], good recoveries were reported in almost all the methods listed in Table 3. However, it has to be emphasized that the validation was usually performed only on spiked samples. In two of the published methods [93,94], authors calculated the recoveries of the method both for spiked and native samples. Recoveries in the native samples were in the range 40%–83% of the recoveries in spiked samples for NSAIDs extraction from sewage sludge [93], but in the same range for SSRIs extraction from the same matrix [94]. Another possible consideration against the use of water as extraction solvent would be the high probability of co-extracting several water-soluble compounds that could interfere with the analysis. This problem is avoided by subsequent LPME, which targets analytes of lower polarity. Yet, co-extracted matrix compounds could interfere with LPME as well. DLLME is more sensitive to the presence of these compounds compared to HF-LPME, in which the fiber effectively shields the solvent and prevents their extraction. In DLLME, one of the possible problems would be the failure of phases to separate after centrifugation because of the emulsifying effect of the co-extractives; in HF-LPME, high-molecular dissolved organic matter from the matrix could adsorb to the fiber and hinder the extraction. However, there are no reports of such problems in the reviewed papers. Generally, chromatograms produced in either GC or HPLC analysis following the LPME were clean and free of interferences. Only a small matrix effect, otherwise a recognized problem in LC-MS with electrospray ionization (ESI), was observed for the extracts prepared by PHWE-HF-LLLME, especially when compared to the established methods, e.g., PLE-SPE [93]. In one method, no chromatographic separation of the extract was used; the extracts obtained by DI-SDME after US extraction of the antibiotic monensin from soil with NaCl solution were directly analyzed by atmospheric pressure- matrix-assisted laser desorption ionization mass spectrometry (AP-MALDI-MS) with very good results [96].

The above discussion and examples given within could serve as a confirmation of the suitability of water-based extraction solutions combined with LPME in the environmental analysis. The common and favorable feature of the sample preparation approaches in this category is the greatly decreased consumption of organic solvents to extract analytes from the solid samples.

**Table 4:** Applications of LPME without previous extraction of solid samples.

LPME	Analytical techn.	Sample	Analytes	Preparation of solid sample	LPME procedure	Optimization of extraction conditions	Method performance	Ref.
DLLME	GC-FPD	soil	chlorpyrifos	"soil solution" ^a^	1.5 mL MeOH (DS) + 40 µL 1-dodecanol (ES) inj. into 25 mL solution at 40 °C; kept still 5 min, added 0.5 g NaCl, shaken; centrif.; ice bath to solidify drop, rinsed with ice w., diss. in 60 µL EtAc	extraction solvent type & *V*, disperser solvent type & *V*, mass of NaCl, extr. time	η 84%–103% (2 levels); RSD < 6.4%; LOD 0.084–0.52 ng/mL ^b^	[99]
US-DLLME	GC-MS	house dust	TBBPA	Kimwipe sprayed with MeOH/AC (1:3), 1 min wiping of 100 cm^2^ area	1 cm^2^ Kimwipe + 800 µL w. + 100 µL MeOH/AC (1:3, DS) + 30 µL ClBz (ES) + 1 drop HCl_conc_ + 50 µL Ac anhydride; US 5 min; centrif.; sedim. phase added Ac anhydr., IS & CH_2_Cl_2_ to 56 µL; US 5 min; heated 60 °C for 5 min, injected	disperser solv. type & *V*, extraction solvent type & *V*, swabbing material	η 104%–106%; RSD < 18%; LOD 2.5 ng/mL; compared with SPE	[100]
HF-LPME	GC-FID	soil	6 PAHs	1 g s.s. + 7 mL AC + 15 mL w.; shaking 30 s	22 mL suspension, 8 µL octane (ES) + IS in 6.5-cm HF; stirring 8 min at 1350 rpm; direct injection	extraction time; HF: stirring rate, *V*_AC_, extr. time, *V*_ES_	η 2.9%–6.2% (PF 80.1–170.7); RSD < 23.3%; LOD 130–220 ng/g	[101]
HF-LPME	HPLC-UV	soil	2 phytohormones	none	5 g s.s. + 14 mL NaCl sol. (340 g/L), 10 µL [BMIM]PF_6_ (ES) in 2.5-cm HF as solvent bar; stirring 50 min at 900 rpm and 25 °C; direct injection	*V*_solv_, extr. time, *T*, NaCl concentration, stirring rate	η 40%–60%; RSD < 7.9%; LOD 5–30 ng/g; compared to ATPS	[88]
HF-LPME	GC-MS	soil	8 triazines	prepared slurry: 20 g s.s./mL w., added NaCl to 10%	3 µL toluene (ES) in 1.3-cm HF in soil slurry; extr. for 20 min at 1000 rpm	extraction solvent, extraction time, stirring rate, addition of NaCl, pH, humic acids	η not given; RSD < 5%; LOD not given; compared with SDME (drop unstable) & SPME (poorer precision)	[102]
HF-LPME	GC-MS	soil	4 chlorophenols	30 mg s.s. + 15 mL w. + IS	15 mL suspension + 15 mL pH 1 buffer sol., 15 µL 1-octanol (ES) in 5.0-cm HF, stirring 80 min at 1100 rpm; direct injection	pH & ionic strength of donor sol., stirring rate, extraction time	η 90.52%–106.47%; RSD < 5.13%; LOD not given for soil; compared with SPME - with HF-LPME less interference	[103]
HF-LPME, dynamic	GC-MS	soil	methylphenols chloro-benzenes, chlorinated pesticides	1 g s.s.+ 4 mL AC/w.(40:60).; US (5 min); stirring at 1000 rpm (40 min)	4 mL suspension, 3 µL toluene (ES) in 1.3-cm HF, stirring 4 min at 200 rpm, dynamic extr. (syringe plunger); direct injection	type & ratio org. solv.: w. in suspension; HF: extr. solvent, extr. time, plunger speed, ionic strength, HA conc.	η 92%–100%; RSD < 13.0%; LOD 50–100 ng/g; comparison with SPME	[104]
HF-LLLME	LC-ESI-MS	sewage sludge	SSRIs	0.25–1 g s.s. + 50 mL w., pH adj. to 12.4	50 mL suspension, DHE in 20-cm HF wall (ES) & 10 µL 0.1 M (NH_4_)H_2_PO_4_ pH 2.1 (AS) in HF lumen; stirring 6 h; direct injection	pH of sample suspension, acceptor solvent & pH, extraction time	η 5%–19% (PF 221-995); RSD < 18.4%; LOD 1–12 ng/g; comparison to HF-LLLME after PHWE	[94]
HF-LLLME	LC-ESI-MS	sewage sludge	4 NSAIDs	0.5–1.5 g s.s. + 50 mL w., stirred 17 h at 660 rpm, pH adj. to 1.5	50 mL slurry, DHE in 18-cm HF wall (ES) & 10 µL 0.1 M (NH_4_)_2_CO_3_ pH 9 (AS) in HF lumen; stirring 4 h at 660 rpm; direct injection	extraction time	η not given; RSD < 17.7%; LOD not given	[105]
DHF-HS-LPME	GC-MS	soil	6 PAHs	1 g s.s. + 1 mL w.; heated at 90 °C for 10 min	3 µL 1-octanol (ES) in 1.5-cm HF in headspace over soil slurry heated at 40 °C for 10 min at 400 rpm, dynamic extr. (5 s dwell time); direct injection	extraction solvent, dwell time, number of cycles, extraction time, *T*, addition of w. & NaCl to s.s.	η not given; RSD < 14.6%; LOD 5.9–76 ng/g	[106]
HS-SDME	GC-FID	drilling mud	C_6_-C_12_ hydrocarbons	drilling mud with water left to separate	5 mL supernatant in vial, a drop (1.5 µL) of *n*-hexadecane (ES) + IS suspended from needle tip in HS; extr. for 30 min at 1000 rpm; direct injection	extraction solvent type & *V*, ionic strength of sample, stirring rate, extraction time	clean chromatograms with no intereferences; other data not given for drilling mud	[107]
HS-LPME	GC-ECD	soil	5 chlorobenzenes	1 g s.s. + 1.5 mL w.; heated for 30 min at 40 °C before extr.	2 µL toluene (ES) into 10-µL microsyringe, 5 µL HS at 40 °C withdrawn at 1µL/s, expelled, 5 s waiting, repeat 25 times; direct injection	extraction solvent type & *V*, HS sampling volume, withdrawal rate, number of cycles	η not given; RSD < 17.7%; LOD 6–14 ng/g; compared with HS-SPME	[44]
CF-SDME (GF-HS-LPME ^c^)	GC-MS	sediment	PAHs	not given	continuous gas flow SDME in a home-designed apparatus: sample heated at 80 °C, extr. for 20 min into 2 µL dodecane (ES) in the gas channel, gas flow rate 2.7 mL/min	gas flow rate, position of solvent drop, i.d. gas outlet channel, extraction time, sample T, extr. solvent T	η not given; RSD < 19.7%; LOD 0.020–8.0 ng ^d^	[108]

^a^ no further details given on preparation; ^b^ LOD given for “soil solution” only; ^c^ name proposed by authors; ^d^ LOD in ng only, sample mass not given. *Abbreviations*: **ES**—extraction solvent; **DS**—disperser solvent in DLLME; **AS**—acceptor solvent in HF-LLLME; **ATPS**—aqueous two-phase system; **DHF**—dynamic hollow fiber; **GF**—gas flow; **IS**—internal standard; **PF**—preconcentration factor; **SPE**—solid-phase extraction; **SPME**—solid phase microextr.; **s.s.**—solid sample; **i.d.**—internal diameter. *Solvents*: **AC**—acetone; **Ac**—acetic; **ClBz**—chlorobenzene; **DHE**—di-*n*-hexyl ether; **EtAc**—ethyl acetate; **MeOH**—methanol; **w.**—water (ultrapure/double deionized/MilliQ); **[BMIm]PF_6_**—1-butyl-3-methylimidazolium hexafluorophosphate.

## 4. LPME without Previous Extraction of Solid or Semisolid Samples

The third category of LPME applied to solid environmental samples presented in this review are the methods of solvent microextraction directly from the solid samples with no previous extraction of analytes with either organic solvent or aqueous solution and no previous separation of the solid sample from the resulting solution. However, some simple pretreatment of the solid sample was still needed. Examples are given in Table 4.

As can readily be seen from Table 4, some minor sample preparation was still needed before the actual LPME. Usually, this just consisted of a slurry preparation from the solid sample with the addition of water [44,88,94,102,103,105,106] or water-organic solvent solution [101,104]. The transfer of analytes into LPME organic solvent was significantly increased from the aqueous slurry compared to the dry solid sample [44]. For some analytes, addition of organic solvent into the suspension, e.g., methanol, markedly increased the recoveries [104]. Other parameters that were commonly optimized in the donor solution were ionic strength [88,102,103,104,106,107] or water-organic solvent solution [101,104], pH [94,102,103] and temperature when LPME from the headspace was performed [44,106,107,108]. Yang *et al.* [108] developed an interesting variation of CF-SDME named gas flow headspace LPME (GF-HS-LPME). A special apparatus was designed in which an aqueous or dry sample was heated, analytes were released into the headspace and transported in a gas flow past the solvent drop positioned in a gas outlet channel. Several parameters were optimized: gas flow rate, position of solvent drop, internal diameter of the outlet channel, temperature of sample and solvent, extraction time. Method was applied to the extraction of PAHs from sediment samples, but unfortunately, no other method characteristics were given for this type of sample except precision and absolute LOD [108]. 

The most frequently applied mode of LPME in this category was HF-LPME [88,94,101,102,103,104,105], mostly with the fiber immersed directly in the suspension and with one solvent only [101,102,103,104] or with an additional acceptor solvent in the fiber lumen [94,105]. This mode of LPME is the most applicable to the aqueous slurry without the previous extraction because the fiber provides the necessary barrier to prevent the sample particles from entering the solvent. Dong *et al.* [88] prepared the fiber filled with ionic liquid as a solvent bar and immersed it into the suspension. Compared to ATPS, performance was slightly poorer, but the solvent (methanol) consumption was decreased from 32 mL to zero per sample [88]. Hou and Lee [104] employed dynamic HF-LPME from the slurry by a moving syringe plunger. Jiang *et al.* [106], however, performed dynamic HF-LPME to extract PAHs from the headspace over the soil slurry heated to 90 °C and got favorable results [106]. Generally, results obtained by the HF-LPME approach were satisfactory and compared well with other techniques: ATPS [88], SPME [102,103,104] or HF-LLLME following PHWE [94]. In the latter case, essentially the same HF-LLLME parameters were applied except for the stirring time, which was shorter with direct HF-LLLME. Both methods have shown very similar performance in terms of LOD and precision [94]. However, some problems were also reported for HF-LPME methods: clogging of the fiber pores with soil particles [101] and decreased extraction efficiency at humic acid concentration above 75 mg/L [104], although Shen and Lee [102] observed no problems at HA concentrations up to 200 mg/L.

Other modes of LPME were less frequently used. One paper was found on DLLME with a solvent lighter than water, where DLLME was also applied on “soil solution” to extract the pesticide chlorpyrifos [99], but as no further details were given on the preparation of this “solution”, one may as well speculate that it was prepared by solvent extraction and therefore the method doesn’t really fall into the present category. Di Napoli-Davis and Owens [100] performed DLLME on an aqueous-methanol-acetone solution in which sorbent material used to wipe dust from electronics surfaces was sonicated. Tetrabromobisphenol-A extracted from the dust was then derivatized and determined by GC-MS. Performance of the method was compared to SPE results: DLLME method gave better precision, recoveries, and LODs [100]. 

Two papers on HS-SDME were found [44,107] apart from CF-SDME method already described [108]. Fang *et al.* [107] extracted C_6_-C_12_ hydrocarbons from the HS of various oil-derived samples, including drilling mud, which was just left to naturally separate and the liquid phase was then extracted [107]. Shen and Lee [44] extracted chlorobenzenes from soil slurry by dynamic in-syringe HS-SDME. Method was compared to HS-SPME and found to have slightly poorer recoveries and LODs [44]. Shen and Lee [102] also designed DI-SDME to extract triazines from soil slurry, but performance was poor because of an unstable solvent drop.

The reported recoveries, RSDs and LODs for the methods listed in Table 4 are generally good and comparable to methods given in Table 2 and Table 3. However, most of the methods in Table 4 were developed primarily for aqueous samples and then applied to some solid sample, therefore the performance data for solid samples are often missing. This fact and a rather small number of publications dealing with this approach in the last decade hint at the possibility that it may not be the best possible from the analytical method performance point of view, although it certainly is from the environmental perspective. 

## 5. Conclusions

Solvent extraction has probably been a mainstay in the isolation of various analytes from very different matrices, including environmental samples for more than a century. Research efforts in the last few decades have been directed towards diminishing its role and importance mainly due to the toxic and environmentally problematic properties of organic solvents. Several new sample preparation techniques have emerged during this time, using minimal quantities of solvents or no solvents at all. However, solvent extraction remains an important sample preparation technique in the environmental analysis of solid environmental samples because of its superior capacity to disrupt the sorption of pollutants to the solid particles and transfer them to the solution amenable to analysis. Therefore, greening efforts in this part of analytical chemistry have gone mainly in the direction of developing and testing more environmentally-friendly solvents or in the direction of miniaturization, which is the subject of the present review. Most established miniaturized liquid-phase extraction techniques are developed for aqueous matrices and the number of publications dealing with LPME applied to solid environmental samples is still very small compared to the number of papers dealing with aqueous samples. In summary, 52 publications dealing with LPME applied on the sample preparation of solid environmental samples have been found and included in this review and categorized into three different groups based on the sample preparation prior to LPME. In many of the methods reviewed in this paper, a particular LPME technique was developed, optimized and validated for aqueous samples and then - more or less marginally - applied to a solid sample. Therefore, many vital data on specific problems arising from the solid matrices, such as realistic recoveries for native samples, aging of the residues, interference from the solid matrices *etc.*, are missing in the majority of the publications. Except in selected papers mentioned in the text, comparison of LPME approach to a more established extraction or clean-up methods is also missing, but would be important for the evaluation of the accuracy of the obtained results. However, there seems to be a positive trend towards introducing LPME techniques in the environmental analysis of solid samples, judging from the number of papers in the last decade. There is certainly a hope that with the number of publications, their methodological quality will also grow, contributing to the greening of yet another segment of analytical chemistry. 
